# Adaptation of the Japanese Version of the 12-Item Attitudes Towards Artificial Intelligence Scale for Medical Trainees: Multicenter Development and Validation Study

**DOI:** 10.2196/81986

**Published:** 2026-01-14

**Authors:** Hirohisa Fujikawa, Hirotake Mori, Kayo Kondo, Yuji Nishizaki, Yuichiro Yano, Toshio Naito

**Affiliations:** 1 Department of General Medicine, Juntendo University Faculty of Medicine, 2-1-1 Hongo, Bunkyo-ku, Tokyo, 113-8421, Japan; 2 Department of Medical Education Studies, International Research Center for Medical Education, Graduate School of Medicine, The University of Tokyo, Bunkyo-ku, Tokyo, Japan; 3 Center for General Medicine Education, School of Medicine, Keio University, Shinjuku-ku, Tokyo, Japan; 4 School of Modern Languages and Cultures, Durham University, Durham, United Kingdom; 5 Division of Medical Education, Juntendo University School of Medicine, Bunkyo-ku, Tokyo, Japan

**Keywords:** artificial intelligence, AI, attitudes toward artificial intelligence, attitudes toward AI, medical education, medical resident, medical student

## Abstract

**Background:**

In the current era of artificial intelligence (AI), use of AI has increased in both clinical practice and medical education. Nevertheless, it is probable that perspectives on the prospects and risks of AI vary among individuals. Given the potential for attitudes toward AI to significantly influence its integration into medical practice and educational initiatives, it is essential to assess these attitudes using a validated tool. The recently developed 12-item Attitudes Towards Artificial Intelligence scale has demonstrated good validity and reliability for the general population, suggesting its potential for extensive use in future studies. However, to our knowledge, there is currently no validated Japanese version of the scale. The lack of a Japanese version hinders research and educational efforts aimed at understanding and improving AI integration into the Japanese health care and medical education system.

**Objective:**

We aimed to develop the Japanese version of the 12-item Attitudes Towards Artificial Intelligence scale (J-ATTARI-12) and investigate whether it is applicable to medical trainees.

**Methods:**

We first translated the original English-language scale into Japanese. To examine its psychometric properties, we then conducted a validation survey by distributing the translated version as an online questionnaire to medical students and residents across Japan from June 2025 to July 2025. We assessed structural validity through factor analysis and convergent validity by computing the Pearson correlation coefficient between the J-ATTARI-12 scores and scores on attitude toward robots. Internal consistency reliability was assessed using Cronbach α values.

**Results:**

We included 326 participants in our analysis. We used a split-half validation approach, with exploratory factor analysis (EFA) on the first half and confirmatory factor analysis on the second half. EFA suggested a 2-factor solution (factor 1: AI anxiety and aversion; factor 2: AI optimism and acceptance). Confirmatory factor analysis revealed that the model fitness indexes of the 2-factor structure suggested by the EFA were good (comparative fit index=0.914 [>0.900]; root mean square error of approximation=0.075 [<0.080]; standardized root mean square residual=0.056 [<0.080]) and superior to those of the 1-factor structure. The value of the Pearson correlation coefficient between the J-ATTARI-12 scores and the attitude toward robots scores was 0.52, which indicated good convergent validity. The Cronbach α for all 12 items was 0.84, which indicated a high level of internal consistency reliability.

**Conclusions:**

We developed and validated the J-ATTARI-12. The developed instrument had good structural validity, convergent validity, and internal consistency reliability for medical trainees. The J-ATTARI-12 is expected to stimulate future studies and educational initiatives that can effectively assess and enhance the integration of AI into clinical practice and medical education systems.

## Introduction

Artificial intelligence (AI) is undergoing rapid development and integration into health care [1,2]. Although the use of AI has raised a number of potential concerns, including a lack of control over the rapidly progressing technology [3], issues of privacy and data protection [4], ethical problems [5], and the potential loss of human jobs [6], the benefits of AI are expected to far outweigh these concerns. AI contributes to improving patient care through enhancing diagnostic accuracy and providing more personalized therapeutic plans [7]. Moreover, AI has the potential to enhance the training and education of medical professionals by offering benefits including virtual simulation and training, remote education, and the recording of teaching videos [8,9]. Consequently, the use of AI has increased in both clinical practice and medical education.

Individuals may hold divergent views on the prospects and risks of AI and adopt varied attitudes toward it [10]. The attitudes of physicians and medical trainees toward AI will have a major impact on its integration into medical practice and educational activities [11]. Negative attitudes toward AI can lead to skepticism and concerns, thereby impeding its adoption [12]. Conversely, positive attitudes toward AI likely foster trust [13], leading individuals to embrace AI systems and amplify their benefits and possibly resulting in their integration into health care [12].

Thus, assessing physicians’ and medical trainees’ attitudes toward AI is critical to identifying potential barriers and fostering acceptance. Accordingly, instruments with robust psychometric properties are required. Although several measures for assessing attitudes toward AI have been developed, only a few have been comprehensively evaluated for reliability and validity. In 2024, Stein et al [10] developed the 12-item Attitudes Towards Artificial Intelligence scale (ATTARI-12). This is a unidimensional scale that integrates affective, behavioral, and cognitive facets into a single measure. The authors subsequently confirmed its psychometric properties for the general population [10], and its wide use in future studies is anticipated.

Despite the potential global applicability of the ATTARI-12, there is currently no validated Japanese version. Japan has a distinct cultural context, and attitudes toward AI are likely influenced by cultural and societal norms. For instance, Japanese culture’s high uncertainty avoidance tends to result in only a gradual adoption of new technologies [14]. In the absence of a culturally adapted measure, assessments may not accurately reflect the true sentiments of Japanese health care workers and trainees. This gap hinders research and educational efforts aimed at understanding and improving AI integration into the Japanese health care system.

In this study, we developed a Japanese version of the ATTARI-12, which was originally created for the general population, and examined whether it is applicable to medical students and resident physicians. We anticipated that the development of this scale in Japan would promote future research and educational courses that effectively assess and improve the integration of AI into clinical practice and the medical education system.

## Methods

This study formed part of a larger research project regarding AI education for medical trainees. We conducted this study in the following 2 steps.

### Step 1: Translation and Cross-Cultural Adaptation

In accordance with an international guideline [15], we translated the original ATTARI-12 into Japanese. First, the first author (HF) asked the creator of the original ATTARI-12 and corresponding author of the article that reported it to allow us to develop the Japanese version. The creator willingly provided permission for our translation of the scale. Second, 2 translators (HF and KK) conducted forward translations independently. Both translators were familiar with Western and Japanese cultures and had rich experience in developing translated versions of scales in the field of health profession education. In particular, KK is a fluent speaker of Japanese and English. Third, the translators performed a synthesis of their translations. Discrepancies were resolved through discussion (version 1). Fourth, HF asked professional bilingual translators who were not involved in our study to translate the Japanese text back into English. HF and KK then compared the back-translated and original English versions item by item and then revised the Japanese version (version 2). Fifth, an expert review of version 2 was conducted by an AI expert (YY) and a health profession education expert (YN) at HF’s request. These experts concluded that no amendment was required. Sixth, HF contacted the creator of the original scale again and asked him to check version 2. The creator concluded that no revision was necessary. Seventh, pilot testing was performed with 2 medical trainees, which indicated no problematic items. Finally, version 2 was confirmed as the finalized Japanese version of the ATTARI-12 (J-ATTARI-12).

### Step 2: Investigation of Psychometric Properties

#### Participants

Between June 2025 and July 2025, we recruited study participants from 5 universities and 9 hospitals across Japan, each of which varied in type and location (Multimedia Appendix 1). We asked the medical education directors of the universities and residency training directors of the hospitals to distribute our anonymous online self-administered questionnaire. An email with a link to the online questionnaire was sent to medical students at the universities and resident physicians at the hospitals via the respective directors. The participants were provided with a brief explanation of the study and indicated their consent to take part by checking the consent box. They were then able to access and complete the questionnaire. The survey duration was 1 month. To maximize the response rate, we sent reminders several times during the survey period.

#### Measures

The original version of the ATTARI-12 has 12 items [10], with responses on a 5-point Likert scale (1=“strongly disagree”; 5=“strongly agree”) [10]. Items 2, 4, 7, 8, 10, and 12 are reverse items and, therefore, are reverse scored [10]. The sum of all item scores is averaged to create a total score ranging from 1 to 5, with higher scores indicating more positive attitudes toward AI.

### Statistical Analysis

To investigate the structural validity of the instrument, we performed exploratory factor analysis (EFA) and confirmatory factor analysis (CFA). Because conducting these 2 types of factor analysis on the same dataset would potentially raise concerns [16], we adopted the split-half validation approach, in which the participants were randomly divided into 2 groups, half (group A) for EFA and the other half (group B) for CFA. As mentioned above, we aimed to develop the scale in a manner that was culturally adapted to the Japanese medical education context. Consequently, EFA was conducted first, followed by CFA.

To determine the appropriateness of EFA, we checked the Kaiser-Meyer-Olkin (KMO) coefficient and Bartlett sphericity test. Running EFA requires a KMO value over 0.80 and a significant result in the Bartlett test [17,18]. We applied EFA to the responses of group A to explore the factor structure of the J-ATTARI-12 using maximum likelihood estimation and Promax rotation. We determined the final factor solution using the results of parallel analysis and the factor loading values (cutoff value=0.30).

We subsequently conducted CFA on group B to confirm the model obtained in EFA. CFA was performed using the maximum likelihood estimator method. Model fitness of CFA is commonly conducted using indexes, including the comparative fit index (CFI), root mean square error of approximation (RMSEA), and standardized root mean square residual (SRMR), with higher CFI (>0.900), lower RMSEA (<0.080), and lower SRMR (<0.080) values indicating a good fit [19-21]. In this study, we compared these indexes between the 2 models, namely, the model suggested by the EFA (2-factor model; described in detail below) and the 1-factor model.

Convergent validity was evaluated through hypothesis testing. On the basis of a previous finding that ATTARI-12 scores were positively linked with specific attitudes toward robots [10], we investigated this relationship using Pearson correlation coefficients. With reference to previous studies [10,22], attitudes toward robots were assessed using 3 items (eg, “Robots are necessary as they can do jobs that are too hard or too dangerous for people”), each of which was answered on a 4-point Likert scale (Multimedia Appendix 2). The scores were computed by averaging the responses to the 3 items (Cronbach α=0.78; mean 3.25, SD 0.55 in our dataset), with higher scores indicating a more positive attitude toward robots. The Pearson correlation coefficients are deemed meaningful if they are greater than 0.30 [23].

We used Cronbach α values to evaluate the internal consistency reliability of the scale. In this study, we computed the values using the responses of the entire sample (N=326; described in detail below). Cronbach α values above 0.70 are acceptable [24].

The possible influence of participant gender and year group on ATTARI-12 scores was explored using descriptive statistics and comparison analyses (independent 2-tailed *t* test or 1-way ANOVA). With regard to gender, as outlined later, the “Others” category comprised only 4 participants, rendering it inadequate for inclusion in a 1-way ANOVA. Given the high likelihood of unstable estimates and heterogeneity of variance associated with such a small group, we compared scores between only the 2 larger groups (man and woman) via independent *t* test. The undergraduate medical curriculum in Japan has traditionally consisted of 2 phases: the first 4 years of preclinical education and the subsequent 2 years of clinical education (ie, clinical clerkship) [25]. Accordingly, medical trainees were divided into preclinical medical students (ie, first- to fourth-year students), clinical medical students (ie, fifth- and sixth-year students), and medical residents. All statistical analyses were performed using R (version 4.5.1; R Foundation for Statistical Computing) and SPSS (version 30.0; IBM Corp), with 2-sided *P* values of <.05 considered statistically significant.

### Ethical Considerations

This study was conducted according to the ethical standards and principles of the Declaration of Helsinki and was approved by the ethics committee of Juntendo University Faculty of Medicine (E25-0028). All participants checked the consent box at the beginning of the questionnaire to indicate their informed consent to take part in the study. To ensure confidentiality, all participant data were anonymized before analysis. Participants were entered into a draw for 1 of 10 ¥5000 (approximately US $30) gift cards.

## Results

### Overview

In total, 9.2% (326/3551) of the eligible participants responded to the survey. There were no missing data. Table 1 shows the participants’ characteristics, and Table 2 shows the responses to each item.

**Table 1.** Characteristics of the participants (N=326).

Characteristic	Participants, n (%)
Gender	
Woman	142 (43.6)
Man	180 (55.2)
Nonbinary	4 (1.2)
Year group	
Medical students	
First	103 (31.6)
Second	19 (5.8)
Third	51 (15.6)
Fourth	60 (18.4)
Fifth	13 (4.0)
Sixth	20 (6.1)
Medical residents	
First	32 (9.8)
Second	28 (8.6)

**Table 2.** Responses to the 12-item Attitudes Towards Artificial Intelligence scale (N=326).

Item (as in the original English-language version)	Responses, n (%)^a^				
	1	2	3	4	5
Item 1: “AI will make this world a better place.”	1 (0.3)	11 (3.4)	72 (22.1)	201 (61.7)	41 (12.6)
Item 2: “I have strong negative emotions about AI.”^b^	49 (15.0)	163 (50.0)	71 (21.8)	37 (11.3)	6 (1.8)
Item 3: “I want to use technologies that rely on AI.”	0 (0.0)	3 (0.9)	36 (11.0)	200 (61.3)	87 (26.7)
Item 4: “AI has more disadvantages than advantages.”^b^	28 (8.6)	171 (52.5)	101 (31.0)	22 (6.7)	4 (1.2)
Item 5: “I look forward to future AI developments.”	2 (0.6)	4 (1.2)	28 (8.6)	157 (48.2)	135 (41.4)
Item 6: “AI offers solutions to many world problems.”	2 (0.6)	24 (7.4)	73 (22.4)	175 (53.7)	52 (16.0)
Item 7: “I prefer technologies that do not feature AI.”^b^	19 (5.8)	124 (38.0)	120 (36.8)	51 (15.6)	12 (3.7)
Item 8: “I am afraid of AI.”^b^	17 (5.2)	62 (19.0)	77 (23.6)	147 (45.1)	23 (7.1)
Item 9: “I would rather choose a technology with AI than one without it.”	5 (1.5)	16 (4.9)	111 (34.0)	154 (47.2)	40 (12.3)
Item 10: “AI creates problems rather than solving them.”^b^	19 (5.8)	115 (35.3)	137 (42.0)	47 (14.4)	8 (2.5)
Item 11: “When I think about AI, I have mostly positive feelings.”	2 (0.6)	45 (13.8)	89 (27.3)	157 (48.2)	22 (6.7)
Item 12: “I would rather avoid technologies that are based on AI.”^b^	38 (11.7)	177 (54.3)	84 (25.8)	23 (7.1)	4 (1.2)

a 5-point Likert scale (1=“strongly disagree” to 5=“strongly agree”)

b These are reverse-scored items.

### Structural Validity Analysis

We performed EFA on group A using maximum likelihood estimation with Promax rotation (154/326, 47.2% of the participants). The KMO value was 0.83, and the Bartlett test was significant (*P*<.001). Table 3 shows the results of the EFA. EFA suggested a 2-factor solution. After discussion among the team members, we named the factors as follows: factor 1 was AI anxiety and aversion, and factor 2 was AI optimism and acceptance.

**Table 3.** Results of the exploratory factor analysis.

	Factor 1	Factor 2
Item 1 factor loading	−0.16	0.73
Item 2 factor loading	0.59	0.18
Item 3 factor loading	0.21	0.52
Item 4 factor loading	0.73	−0.07
Item 5 factor loading	−0.05	0.85
Item 6 factor loading	0.05	0.35
Item 7 factor loading	0.73	−0.06
Item 8 factor loading	0.30	0.12
Item 9 factor loading	−0.05	0.47
Item 10 factor loading	0.78	−0.15
Item 11 factor loading	0.12	0.44
Item 12 factor loading	0.64	0.07
Eigenvalue	3.79	0.76
Percentage of the variance explained	22	18

**Figure 1.**
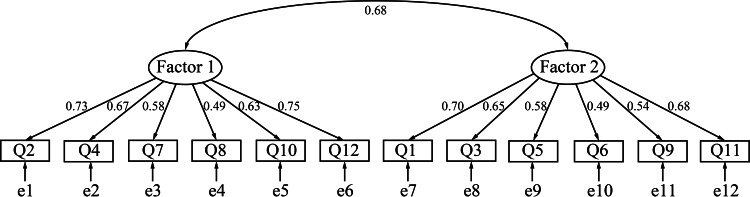
Factor structure of the Japanese version of the 12-item Attitudes Towards Artificial Intelligence scale (confirmatory factor analysis). Ellipses indicate latent variables (factors). Rectangles indicate observed variables (items). Values on single-headed arrows indicate standardized factor loadings. Values on double-headed arrows indicate correlation coefficients.

We then performed CFA on group B (the remaining 172/326, 52.8% of the participants) using the maximum likelihood estimator method. The 2-factor model suggested by the EFA yielded better goodness-of-fit results (CFI=0.914; RMSEA=0.075; SRMR=0.056) than the 1-factor model (CFI=0.804; RMSEA=0.113; SRMR=0.078). Multimedia Appendix 3 shows the CIs for the RMSEA. Figure 1 shows the path diagram. Therefore, we adopted the 2-factor model.

### Convergent Validity Analysis

We computed the Pearson correlation coefficient between the ATTARI-12 scores and the attitude toward robots scores. The coefficient value was 0.52 (*P*<.001), which indicated a positive correlation between these 2 scores.

### Internal Consistency Reliability Analysis and Descriptive Statistics

Table 4 shows the descriptive statistics with the Cronbach α values. The Cronbach α for all 12 items was 0.84. The Cronbach α values for factors 1 and 2 were 0.80 and 0.76, respectively. These values were above the cutoff (0.70).

**Table 4.** Internal consistency reliability analysis and descriptive statistics.

	Number of items	Mean (SD)	Observed range	Cronbach α
Total	12	3.61 (0.50)	2.08‐5.00	0.84
Factor 1	6	3.36 (0.63)	1.00‐5.00	0.80
Factor 2	6	3.87 (0.52)	2.17‐5.00	0.76

Table 5 shows descriptive data and comparison by gender and year group. The comparison analyses did not show any significant differences in ATTARI-12 scores.

**Table 5.** Descriptive data and comparison by gender and year group.

	Score on ATTARI-12^a^, mean (SD)	*P* value
Gender^b^		.71^c^
Woman	3.60 (0.47)	
Man	3.62 (0.53)	
Year group		.21^d^
First- to fourth-year medical students	3.58 (0.51)	
Fifth- and sixth-year medical students	3.67 (0.55)	
Medical residents	3.70 (0.42)	

a ATTARI-12: 12-item Attitudes Towards Artificial Intelligence scale.

b The “Nonbinary” group included only 4 participants, which is too small to be appropriately included in a 1-way ANOVA. Given the high likelihood of unstable estimates and heterogeneity of variance associated with such a small group, we compared scores between only the 2 larger groups (man and woman).

c *P* value from independent-sample *t* test.

d *P* value from 1-way ANOVA.

## Discussion

### Principal Findings

In this study, we translated the ATTARI-12, originally developed for the general population, into Japanese in accordance with an international guideline [15] and then validated its structural and convergent validity and internal consistency reliability for medical trainees. Applying this scale in the context of medical trainees in Japan has the potential to stimulate future research and educational interventions. Such initiatives would serve to effectively assess and enhance the integration of AI in clinical practice and the medical education system.

Our study found that the internal consistency reliability of the J-ATTARI-12 was good. The findings were consistent with those of the original ATTARI-12 developmental study [10]. The original study reported Cronbach α values of 0.93 for a US sample and 0.90 for a German sample [10], which suggested that the ATTARI-12 is likely a helpful measure with good internal consistency reliability across countries.

Factor analysis indicated that the J-ATTARI-12 had a 2-factor structure, in contrast to the original English-language version’s unidimensional structure. The original ATTARI-12 was conceptualized as a unidimensional scale. The original article by Stein et al [10] suggested that the developers intentionally balanced positively and negatively worded items. This design makes it unsurprising that the Japanese version yielded 2 factors corresponding to negative (factor 1: AI anxiety and aversion) and positive (factor 2: AI optimism and acceptance) item valence. Therefore, we should acknowledge that, although 2 factors emerged, they simply reflect positive versus negative wording and that, for the purpose of international comparison, using a total score based on a 1-factor assumption may remain preferable. At the same time, the content of the 2 factors (ie, AI anxiety and aversion vs AI optimism and acceptance) may reflect attitudinal domains that are influenced by broader cultural characteristics, such as uncertainty avoidance, technophilia, or collectivism. To determine whether a 2-factor structure offers conceptual or psychometric advantages beyond a unidimensional model and whether this pattern is observed across different cultural contexts, future cross-cultural validation studies with larger and more diverse samples are required.

We note a couple of potential limitations of our study. First, the response rate was relatively low. In addition, the survey was conducted using convenience sampling and included only 14 institutions, and it is likely that participating institutions or individuals had a greater interest in AI. It is increasingly challenging to obtain high response rates to online surveys, and rates frequently drop to 10% [26,27]. Nevertheless, the literature suggests that the response rate to our survey may have been sufficient to provide reliable data [28,29]. Additionally, it should be noted that, despite the absence of accurate statistics on the demographic variables of medical trainees across Japan, recent reports have indicated that the proportion of female physicians aged ≤29 years stands at approximately 40%, with a steady upward trend observed in recent years [30]. Thus, the finding that 43.6% (142/326) of the respondents were women does not generate concerns regarding the representativeness of the sample. Second, in this study, although we evaluated the structural validity, convergent validity, and internal consistency reliability of the scale, other determinants of validity (eg, discriminant validity and predictive validity) and reliability (eg, test-retest reliability) have yet to be examined. Future studies should examine these psychometric properties. Third, the use of a gift card lottery can raise concerns about bias. Nevertheless, this method is commonly used and appears to be acceptable [31-34]. Fourth, our results may indicate a ceiling effect. For example, 41.4% (135/326) of the respondents selected the highest score (5) for item 5. However, according to the original ATTARI-12 paper, responses tend to be skewed toward a score of 4, suggesting that the scale inherently elicits generally positive attitudes toward AI. Therefore, these findings do not represent an unusual deviation but are consistent with the response patterns reported in the original study.

Despite these limitations, this study produced the first Japanese version of the ATTARI-12, which is likely to be used as a novel measure for assessing AI attitudes among medical students and residents. Two outcomes are expected. First, by incorporating the J-ATTARI-12 into medical curricula, medical educators will be able to develop a more comprehensive understanding of the trainees’ readiness for AI adoption. This will likely be advantageous in the design of customized educational interventions using AI based on the level of each trainee’s attitude toward AI [35] and will support curriculum development by enabling medical educators to identify learners who may need more foundational AI exposure or targeted support. Second, the J-ATTARI-12 will facilitate medical education research in Japan. The scale allows researchers to examine the impact of specific educational interventions on attitude change concerning AI, and it is suitable for repeated administration in longitudinal studies to track how attitudes evolve over the course of training. In addition, the availability of the J-ATTARI-12 will promote cross-cultural comparison of attitudes toward AI and its possible outcomes internationally. For these reasons, we expect that the J-ATTARI-12 will play a pivotal role in facilitating data-informed curriculum development and contribute to the expanding body of medical education research in this era of AI.

### Conclusions

In this study, we developed the J-ATTARI-12, originally developed for the general population, in accordance with an international guideline. A validation survey revealed that the structural and convergent validity, as well as the internal consistency reliability, were good for medical trainees in Japan. The developed measure can be used for customized educational initiatives using AI based on the level of each trainee’s attitude toward AI. It will also provide helpful information to medical education researchers in Japan in this era of AI.

## Supplementary material

10.2196/81986 Multimedia Appendix 1 Characteristics of the participating institutions.

10.2196/81986 Multimedia Appendix 2 Measure for attitudes toward robots.

10.2196/81986 Multimedia Appendix 3 CIs for the root mean square error of approximation.

## Abbreviations

AI: artificial intelligence
ATTARI-12: 12-item Attitudes Towards Artificial Intelligence scale
CFA: confirmatory factor analysis
CFI: comparative fit index
EFA: exploratory factor analysis
J-ATTARI-12: Japanese version of the 12-item Attitudes Towards Artificial Intelligence scale
KMO: Kaiser-Meyer-Olkin
RMSEA: root mean square error of approximation
SRMR: standardized root mean square residual
