# Supplementary material for: Adaptation of the Japanese Version of the 12-Item Attitudes Towards Artificial Intelligence Scale for Medical Trainees: Multicenter Development and Validation Study
Source: JMIR Med Educ. 2026 Jan 14;12:e81986. doi: 10.2196/81986 (PMC12808871; doi:10.2196/81986)
Supplement: Multimedia Appendix 1 [file mededu-v12-e81986-s001.docx]

**Multimedia Appendix 1: Characteristics of the participating institutions^a^**

|  | **N (%)** |
| --- | --- |
| **University (medical students)** |  |
| Type  National university  Private university | 2 (40)  3 (60) |
| Location  Kanto  Chubu  Kinki  Kyushu | 2 (40)  1 (20)  1 (20)  1 (20) |
| **Hospital (medical residents)** |  |
| Type  Community hospital  University branch hospital  University hospital | 4 (44)  3 (33)  2 (22) |
| Location  Hokkaido and Tohoku  Kanto  Chubu  Kyushu | 1 (11)  4 (44)  3 (33)  1 (11) |

^a^ 5 universities and 9 hospitals participated in the study.
