# Supplementary material for: Adaptation of the Japanese Version of the 12-Item Attitudes Towards Artificial Intelligence Scale for Medical Trainees: Multicenter Development and Validation Study
Source: JMIR Med Educ. 2026 Jan 14;12:e81986. doi: 10.2196/81986 (PMC12808871; doi:10.2196/81986)
Supplement: Multimedia Appendix 2 [file mededu-v12-e81986-s002.docx]

**Multimedia Appendix 2: Measure for attitudes towards robots**

Now we are interested in your evaluation of robots. A robot is a machine which can assist humans in everyday tasks without constant guidance or instruction, e.g. as a kind of co-worker helping on the factory floor or as a robot cleaner, or in activities which may be dangerous for humans, like search and rescue in disasters.

[Item 1] Generally speaking, do you have a very positive, fairly positive, fairly negative or very negative view of robots? (from 0 = very negative to 3 = very positive)

[Items 2 and 3] Please indicate below how much you agree with the following statements. (from 0 = totally disagree to 3 = totally agree)

“Robots are a good thing for societies because they help people.”

“Robots are necessary as they can do jobs that are too hard or too dangerous for people.”
