# Supplementary material for: Adaptation of the Japanese Version of the 12-Item Attitudes Towards Artificial Intelligence Scale for Medical Trainees: Multicenter Development and Validation Study
Source: JMIR Med Educ. 2026 Jan 14;12:e81986. doi: 10.2196/81986 (PMC12808871; doi:10.2196/81986)
Supplement: Multimedia Appendix 3 [file mededu-v12-e81986-s003.docx]

**Multimedia Appendix 3: Confidence intervals for root mean square error of approximation**

| **Model** | **RMSEA** | **LLCI** | **ULCI** |
| --- | --- | --- | --- |
| Two-factor model | 0.075 | 0.054 | 0.097 |
| One-factor model | 0.113 | 0.094 | 0.132 |

Abbreviations: CI, confidence interval; LLCI, lower level confidence interval; RMSEA, root mean square error of approximation; ULCI, upper level confidence interval
